# A Passive Wireless Temperature Sensor for Harsh Environment Applications

**DOI:** 10.3390/s8127982

**Published:** 2008-12-08

**Authors:** Ya Wang, Yi Jia, Qiushui Chen, Yanyun Wang

**Affiliations:** 1 Department of Mechanical Engineering, University of Puerto Rico - Mayagüez Campus, Mayagüez, Puerto Rico, 00681-9045, USA; 2 Boston Applied Technologies, Inc., 6F Gill Street, Woburn, MA 01801, USA

**Keywords:** High Temperature Sensor, Hash Environment Applications, Passive, Wireless

## Abstract

High temperature sensors capable of operating in harsh environments are needed in order to prevent disasters caused by structural or system functional failures due to increasing temperatures. Most existing temperature sensors do not satisfy the needs because they require either physical contact or a battery power supply for signal communication, and furthermore, neither of them can withstand high temperatures nor rotating applications. This paper presents a novel passive wireless temperature sensor, suitable for working in harsh environments for high temperature rotating component monitoring. A completely passive *LC* resonant telemetry scheme, relying on a frequency variation output, which has been applied successfully in pressure, humidity and chemical measurement, is integrated with a unique high-k temperature sensitive ceramic material, in order to measure the temperatures without contacts, active elements, or power supplies within the sensor. In this paper, the high temperature sensor design and performance analysis are conducted based on mechanical and electrical modeling, in order to maximize the sensing distance, the *Q* factor and the sensitivity. In the end, the sensor prototype is fabricated and calibrated successfully up to 235°C, so that the concept of temperature sensing through passive wireless communication is proved.

## 1. Introduction

In order to prevent disasters caused by structural failure due to high temperatures, sensors capable of measuring high temperatures in harsh environments are needed, for example, for high temperature monitoring of heat resistant tiles of the space shuttle, high temperature testing of rotating bearings in the aircraft engine, and qualification testing of disc brakes, jet engine dynamics and high-speed shaft rotations [1-4].

Existing high temperature sensing devices are briefly introduced in this section, which include high temperature thermocouples, high temperature optical sensors, high temperature surface acoustic sensors *(SAW)*, as well as the *RF* powered *LC* sensors.

A thermocouple is an assembly of two wires of different metals joined at one end, hot end, for temperature measuring, and at the _other_ end, the cold junction, that usually works as a reference at 0°C. Some available commercial high temperature thermocouples are even able to measure temperature up to 2300°C. However, the signal outputting from a thermocouple is weak and can easily be affected by common mode noises. Furthermore, they drift significantly under high temperature environments during long-term operation [5]. In some hostile environments, they have a limited life of only a few days because of their susceptibility to attack from corrosive chemicals.

High optical temperature sensors are instruments converting external temperature stimulus optical signals, characterized by several variables, such as intensity, spectrum, phase and state of polarization. The main existing techniques for optical thermometry measure temperatures by detecting the thermal radiation emitted by the object [6] or the change in optical path length of a short piece of material whose thermal expansion coefficient and refractive index change as a function of temperature [7]; or by detecting the decay time or intensity of a *UV* stimulated visible fluorescence pulse, which is temperature dependent [8], or based on temperature dependent optical scatterings [9]. Most of these optical sensors offer several significant advantages, such as small size, light weight and some of them can even test temperature up to 1,500°C [7]. However, these commonly used traditional techniques are difficult to apply for measurements in hostile environments, e.g., electromagnetic interference, radiation, corrosion and rotating components, where performance attainable in measurement sensitivity, accuracy and range is limited.

High temperature Surface Acoustic Wave *(SAW)* sensors are based on detecting the change in phase velocity of the surface acoustic wave caused by temperatures [10]. The velocity change can be monitored by measuring the frequency or phase characteristics of the sensor and then can be correlated to the corresponding temperature quantity being measured. In fact, all acoustic wave devices are piezoelectric acoustic wave sensors, applying an oscillating electric field to create a mechanical wave, which propagates through the substrate and is then converted back to an electric field for measurement. This technology is especially useful for some harsh environments, where it is difficult to insert probes for measuring temperature due to low thermal mass, low conductivity, or the strong radiation coupling of the components at high temperatures. However, as the acoustic wave propagates through or on the surface of the material, any changes to the characteristics of the propagation path affect the velocity and/or amplitude of the wave. Hence, the main difficulty with this technique is that the speed of sound is strongly dependent on not only temperature, but also environmental, geometric and material properties along the path, which is usually changed in different environmental conditions. The effects yield low system capacity, low bandwidth and limits the range of application.

Dozens of patens and papers have been published related to the application of *RF* powered *LC* sensors in the last decade, because these sensors eliminates the need for onboard power and exposed interconnections. As we know, a battery is usually the element that limits the sensor lifetime and the operating temperature range, and “virtual batteries” powered by *RF* radiation is a promising alternative to chemical batteries. Because their small size and stable characterization, the *RF* powered *LC* sensors are particularly suitable for transmitting high energy for short distances as required in harsh medical and industrial environments, where, for instance, high temperature pressure sensors [11-13], high temperature chemical sensors [14], and humidity sensors [15] etc. This low frequency range generally allows the use of higher data transfer rates, which is required for reading fast sensor response or reading of multiple sensor modules [16]. They have made significant contributions to advance the *LC* based passive wireless sensing technologies and extend their applications in many areas, such as pressure, humidity and chemical measurements.

**Figure 1.**
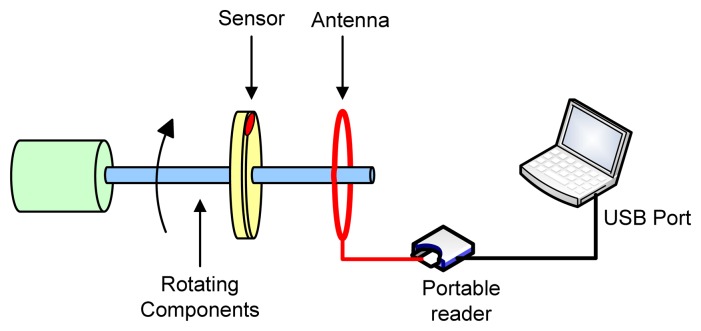
Proposed Wireless Temperature Sensing System.

However, the development of the *RF* powered *LC* temperature sensor hasn't been demonstrated so far for harsh environment application up to 235°C. In this paper, a novel *RF* powered *LC* temperature sensor is developed, capable of operating in harsh environments for high temperature rotating component monitoring. As illustrated in Figure 1. The temperature information is detected by the portable reader across antenna and then transmitted to Laptop. This project extended the knowledge base of passive wireless temperature sensing technologies in harsh environment and advance embedded prognostics health monitoring technologies. The uniqueness and novelties of the research are to:

1) apply the unique high-k temperature sensitive ceramic material instead of any electronic components to realize *LC* tank temperature sensing;
2) develop a novel high-k temperature sensitive ceramic material capable of working in harsh environment up to 235°C;
3) integrate the unique temperature sensitive ceramic material into the *LC* resonant tank circuit to create a frequency-encoded sensing mechanism which could dramatically increase sensitivity and dynamic response of the wireless temperature sensor.

In Section 2, the novel wireless passive temperature sensor design and simulation is presented, based on mechanical and electrical modeling. A completely passive *LC* resonant telemetry scheme, which has been applied successfully in pressure [11-13], chemical [14] and humidity [15] measurement, is integrated with the high-k temperature sensitive ceramic material, in this paper, to measure the changing temperatures, removing contacts, active elements, or power supplies to be contained within the sensor. In Section 3, performance analysis is conducted to determine sensor size, resonant frequency range, to maximize sensing distance, *Q* factor and sensitivity. At last, in Section 4, the sensor prototype is successfully fabricated and calibrated up to 235°C, so that the concept of temperature sensing through passive wireless communication is proved. Conclusions are consequently presented in Section 5.

## 2. Sensor Design and Simulation

**Figure 2.**
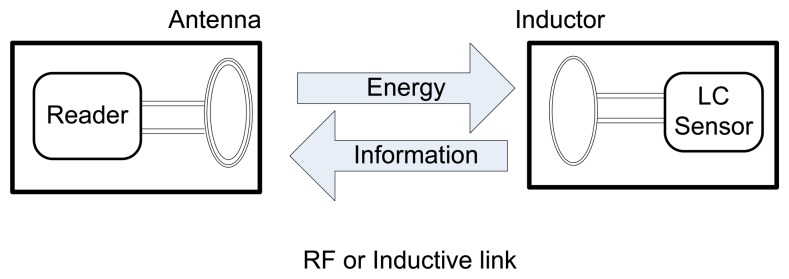
Basic Schematic of the Wireless Communication Proposed.

The wireless interrogation and remote powering is achieved through an inductively powered system which generates a time varying electromagnetic field based on Faraday's law of induction and Lenz's law. As shown in Figure 2, the temperature sensor is powered by a remote reader which sends out an oscillating magnetic field by an inductive link between the reader's antenna and the sensor's inductor. At the same time, the temperature information in terms of a resonant frequency is also transmitted to the reader side through this inductive link. Because the resonant frequency of the remote reader changes when the capacitance of the sensor changes in response to environmental variables “temperature”, the remote reader can detect frequency variations in the sensor's response by monitoring the impedance across the terminals of the wide bandwidth reader antenna.

**Figure 3.**
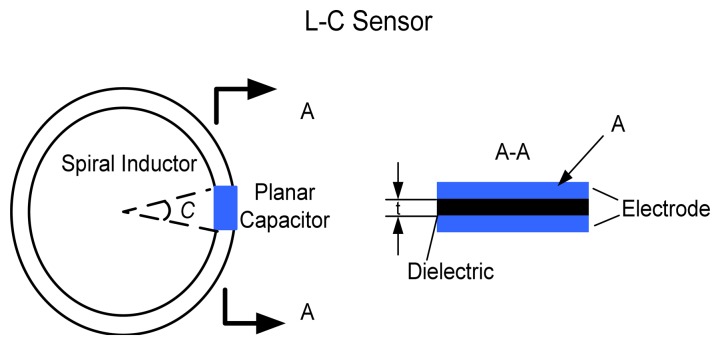
Schematic Diagram of Sensor Design.

The temperature sensor, as demonstrated in Figure 3, is composed of a ceramic multi-layer capacitor integrated with planar inductor, which forms an *LC* resonant circuit. This planar capacitor structural design, incorporating with thick film high-k temperature sensitive ceramic material and thick film electrode, makes the sensor easy to attach and can be used on round rotating components, like bearings in the aircraft engine.

Electrical Capacitance (*EC*) is the basic design principle of this temperature sensor. The capacitance of the sensor is a function of the dielectric constant of the sensitive material. The planar capacitor, when exposed to harsh environments, has a linear dielectric constant variation with the temperature. The capacitance changes in response to temperature can be expressed in equation (1).

(1) $$C_{S}(T)=\frac{{ɛ}_{0}{ɛ}_{r}(T)A}{t}$$

Here *ε*_*0*□_ is the permittivity of free space of 8.85 × 10^-12^ *F*/*m* and *ε_r_* corresponds to the relative dielectric constant of the dielectric material. *A* indicates the area of the electrode plate, and *t* is the distance between the electrode plates, which is also the thickness of dielectric material. Once the sensor is exposed to changing temperatures, a dielectric constant variation in the material *ε_r_* (*T*) will occur.

**Figure 4.**
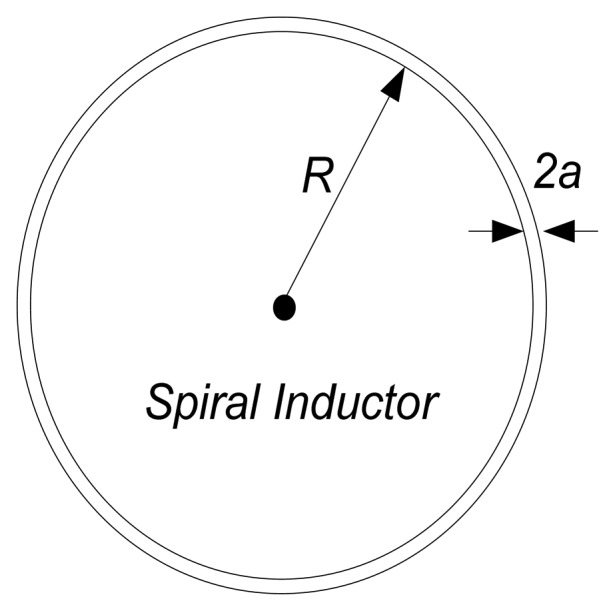
Spiral Inductor Design.

The spiral inductor, as illustrated in Figure 4, constitutes a *LC* resonant circuit element for the temperature sensor, which provides a high quality factor element, feasible in harsh environment applications. Coupling the primary reader antennal (in Figure 2), the spiral inductor acts as a transformer based on the principle of electromagnetic induction. When an oscillating current is executed on the reader antenna, a changing magnetic field to both the primary and sensor inductor is produced along the magnetic path in the air. An alternating voltage of the same frequency is induced in the sensor inductor. The environmental temperature variations induce the frequency change, which can be detected from reader side by monitoring the impedance across the terminals of the wide bandwidth reader antenna. In other words, the electrical energy is transferred from the input coil to the sensor and at the same time, the temperature information is detected from the reader across the coupled magnetic field.

In order to read out the temperature information, it is necessary to design and fabricate an appropriate inductor to have both a reasonable inductance and a quality factor at high temperatures. Actually, there is no closed-form solution for the inductance of circular spiral inductors. The self-inductance of a circular loop of round wire (in Figure 3) has a low frequency inductance that can be estimated by [17]:

(2) $$L_{s}\approx n^{2}\mu_{0}R[\ln(\frac{8R}{a})-1.75]$$

Here *n* means the inductor turns, *R* indicates the loop radius*, a* corresponds to the wire radius, and the magnetic permeability of free space *μ_0_* is 4π × 1-^-7^*H*/*m*.

The resonant frequency of the sensor indicates the point where a sudden change appears in the frequency response of the impedance. The expression of the resonant frequency is defined by the following equation:

(3) $$f_{r}(T)=\frac{1}{2\pi \sqrt{L_{s}C_{s}(T)}}$$

**Figure 5.**
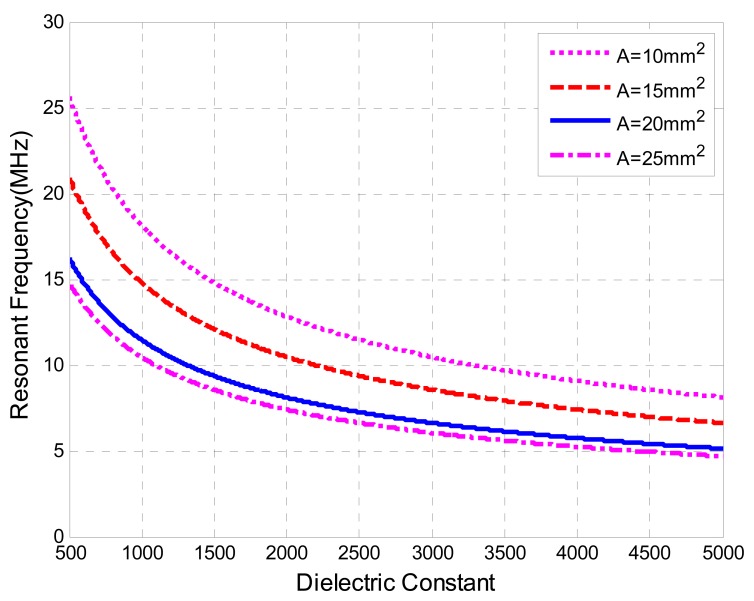
the Frequency *vs.* Dielectric Constant.

The simulation has been performed to present a general idea of relationship between the resonant frequency and dielectric constant, as illustrated in Figure 5, for a sensing area of electrode plate, *A*, ranging from *10 mm^2^* to *25 mm^2^*, a thickness of the sensitive material, *t*, of *0.480 mm*, a wire radius, *a*, of *0.337 mm*, an inductor radius, *R*, of *8.5 mm*, and turns of the inductor, *n*, of *2*.

## 3. Performance Analysis

### 3.1 Electrical Model

**Figure 6.**
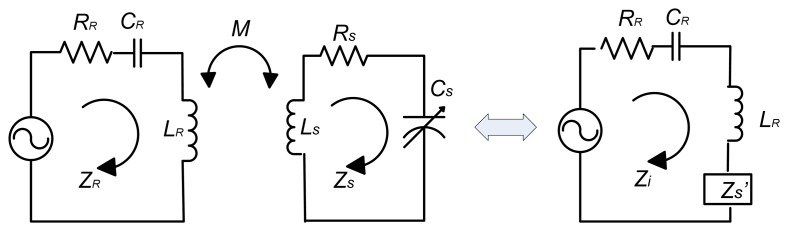
Equivalent Circuit Diagram of Wireless Telemetry System.

The classical approach to analyze the wireless sensor system is to eliminate the coupled reader coil and the sensor by reflecting impedances back to the reader, in order to clarify the voltage change of reader coil. The equivalent circuit diagram is shown in Figure 6. Here, *Z_R_* and *Z_S_* indicates the inherent impedances from the reader and sensor respectively, *R_R_* and *R_S_* are the self resistances of the reader and the sensor circuit, *C_R_* is the capacitance of the reader circuit, which is introduced in order to maximize the current applied through the reader antenna, *C_S_* is the sensor capacitance, which is sensitive to environment temperature. *Z_s_'* is the reflected impedance of the sensor, *Z_i_* is the input impedance seen from the reader side, and *M* corresponds to the mutual inductance.

The impedance of the resonant circuit of the reader is given by:

(4) $$Z_{R}=j\omega L_{R}+R_{R}+\frac{1}{j\omega C_{R}}$$

The impedance of the sensor side can be expressed as:

(5) $$Z_{s}^{\prime }=\frac{{\left(\omega M\right)}^{2}}{j\omega L_{S}+R_{S}+\frac{1}{j\omega C_{S}}}=-\frac{\omega^{2}k^{2}L_{R}L_{S}}{j\omega L_{S}+R_{S}+\frac{1}{j\omega C_{S}}}$$

Here *k* is the coupling coefficient, defined by:

(6) $$k=\frac{M}{\sqrt{L_{R}L_{S}}}$$

In fact, the sensor interaction can be seen as a load Δ *Zs*' placed in series with the inductance antenna:

(7) $$\Delta {Z_{S}}^{\prime }=\frac{\omega^{2}k^{2}L_{R}L_{S}}{j\omega L_{S}+R_{S}+\frac{1}{j\omega (C_{S}+\Delta C_{S})}}$$

Therefore, the input impedance seen from the reader side is given by

(8) $$Z_{i}=Z_{R}+Z_{S}^{\prime }$$

Substitute (4) and (7) into (8), it will yield:

(9) $$Z_{i}=j\omega L_{R}+R_{R}+\frac{1}{j\omega C_{R}}+\frac{\omega^{2}k^{2}L_{R}L_{S}}{j\omega L_{S}+R_{S}+\frac{1}{j\omega (C_{S}+\Delta C_{S})}}$$

**Figure 7.**
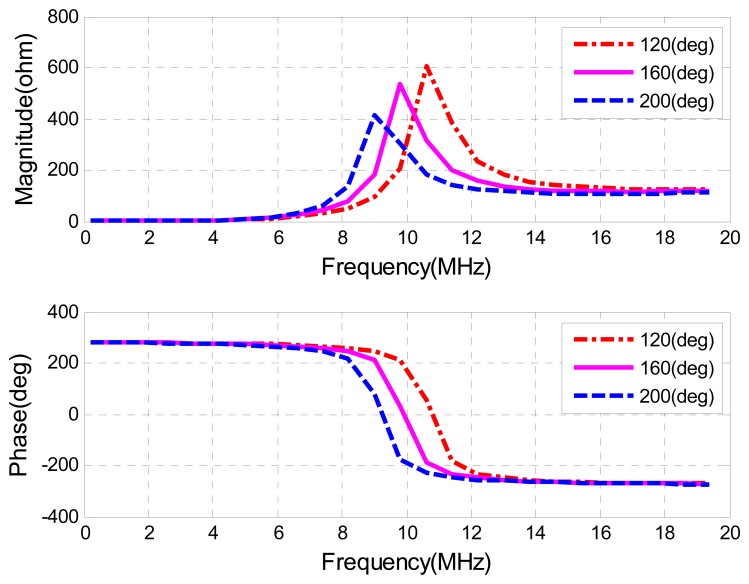
Input Impedance *vs.* Sweeping Frequency.

A voltage drop caused by the sensor side circuit is treated as the reflected impedance *Z_S_*' multiplied by the current. Actually, instead of measuring the voltage change at a certain frequency, a periodical sweeping frequency around the sensor's natural frequency is generated to measure a frequency variation. As the reader's frequency is swept, once the excitation frequency matches to the resonant frequency from the sensor side, a sudden increase in the sensor impedance *Z_S_*' occurs. Figure 7 shows the magnitude of impedance and phase of this reflected sensor impedance *Z_S_*' verse the sweeping frequency changes at different temperatures, with the parameter values summarized in Table 1.

In order to be able to detect the resonant frequency from the reader's side, the variation of the reflected impedance, *Zs*', should be high enough. If the changes of reflected impedance from the sensor, *Zs*', are smaller than those of inductance from the antenna, most of the voltage drop will occur across the antenna. This will bury the change of reflected impedance *Zs*' caused by the remote sensor. For this reason, the *Q*-factor and coupling coefficient should be high enough to make sure that the resonant frequency can be detected.

### 3.2 Q-factor

The quality factor, *Q*, is defined as the ratio of total stored energy to dissipated energy per unit cycle of the resonant system:

(10) $$Q=\frac{W_{\text{total}}}{P/2\pi f}=\frac{2\pi fW_{\text{total}}}{P}$$

where *W* corresponds to the total stored energy, *P* indicates the average dissipated power, and *f* is the resonant frequency. There is another expression of *Q*-factor defined as follow:

(11) $$Q=\frac{2\pi f_{r}L_{S}}{R_{L}}=\frac{1}{R_{L}}\sqrt{\frac{L_{S}}{C_{S}}}$$

where *R_L_* is the total sensor resistance consisting of the inductor resistance, capacitor resistance and circuit resistance, which is briefly discussed by Musunuri et. al. in [18].

**Figure 8.**
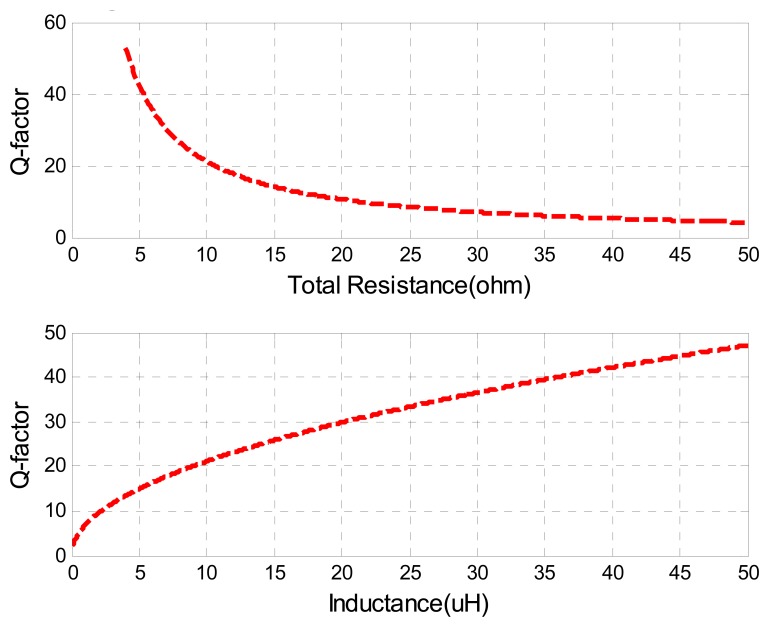
Q-factor *vs.* Resistance & Inductance.

Qualitatively, the *Q*-factor is generally interpreted as an indication of the sharpness of the resonance peak. A higher *Q*-factor indicates a lower rate of energy dissipation relative to the oscillation frequency. Figure 8 presents the simulation of the *Q*-factor verses total sensor resistance and sensor inductance, where the same nominal values of other parameters in Table 1 are used.

### 3.3 Coupling Factor

The coupling coefficient *k* is the factor dominating the wireless range of inductive telemetry systems. For a complete system description, *k* can be modeled and optimized specifically for each application by using finite element *CAD* tools and models. However, a fair approximation of *k* related to design parameters is given by [19]:

(11) $$k(d)={\left(\frac{r_{S}r_{R}}{d^{2}+r^{2}}\right)}^{\frac{3}{2}}$$

**Figure 9.**
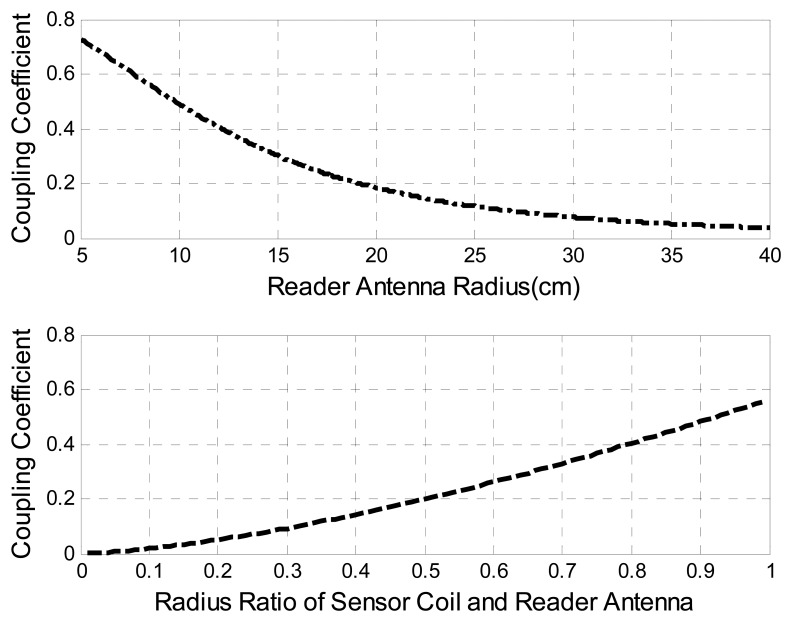
Coupling Coefficient *vs.* Reader Antenna Radius & Radius Ratio.

Here *r_R_* corresponds to the radius of the primary coil, *r_S_* indicates the radius of the sensor inductor, and *d* means the coupling distance. Figure 9 presents how the reader antenna radius and the radius ratio of the sensor radius and the reader antenna affect the coupling factor *k* at a certain communication distance (parameters used are shown in Table.1) The simulation indicates that minimizing the antenna radius and maximizing the radius ratio will improve the coupling factor and maximize the coupling distance.

### 3.4 Discussions

In this section, the performance investigation of the passive wireless temperature sensor is conducted with respect to resonant frequency, *Q*-factor and coupling factor. In addition to the parameters adjustment mentioned above, capacitance compensation can also be adopted to maximize reflected sensor impedance in order to make the resonant frequency detectable, that is, introducing a capacitance into the reader's side to make another resonant circuit, leaving the full sensor impedance change expressed at the reader's side. However, this capacitor would be able to change according to the resonant frequency value for maximum efficiency, which may not be easily accessible. This would also require more sophisticated compensation architectures.

All in all, improvements are concluded as follows in order to achieve high performance wireless system, characterized with a high *Q*-factor and a maximized communication distance:

1) to maximize sensor inductance, the radius ratio of the sensor inductor and reader coil;
2) to minimize the sensor resistance, the radius of the reader antenna, reader inductance;
3) to introduce reader capacitance compensation.

## 4. Sensor Calibration

**Figure 10.**
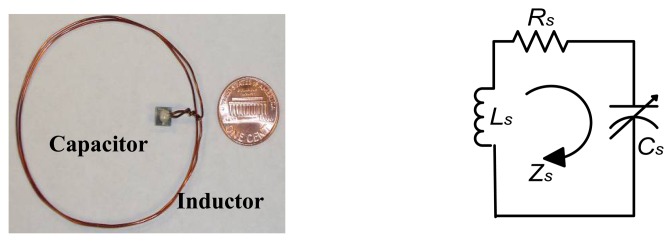
the Sensor Prototype and Equivalent Circuit.

The sensor prototype and its equivalent circuit are shown in Figure 10. The spiral inductor with two turns is made of enamel-coated magnet copper wire, connected to the electrodes of the capacitor by *6041D* Ag. The diameter of the round copper wire *2a* is *0.674 mm*; the radius of the inductor *R* is *28.5 mm*. The area of the electrode plate *A* is *25 mm^2^* and the thickness of dielectric material *t* is *0.480 mm*. Here, *L_S_* indicates the sensor inductance, *C_S_* means the sensor capacitor, *R_S_* corresponds to the ohmic resistance in the sensor and Z*_S_* demonstrates the impedance of this sensor prototype.

**Figure 11.**
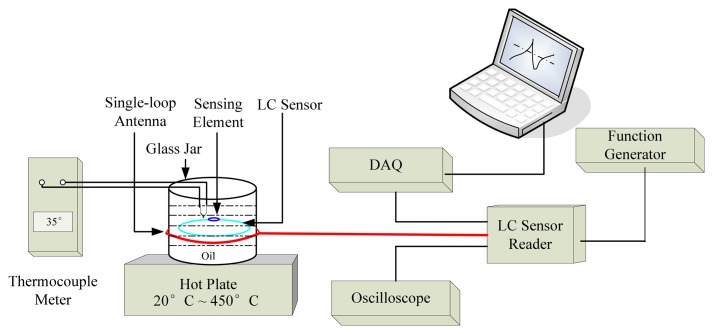
Principle of the Sensor Calibration Experiment.

Figure 11 demonstrates the principle of sensor calibration experiment. The temperature sensor is deposed into a glass of oil, which is heated using Hot Plat, providing the temperature range from room temperature to 400 °C. The oil's temperature is measured by thermometers directly. At the same time, the antenna of the reader is laid outside of the glass jar, which transfers the temperature information to the reader. This information can be read out from Oscilloscope in terms of Resonant Frequency. Moreover, the *DAQ* card can also acquire the temperature variation information in real-time and then be read out from laptop using *Matlab* data acquisition toolbox.

**Figure 12.**
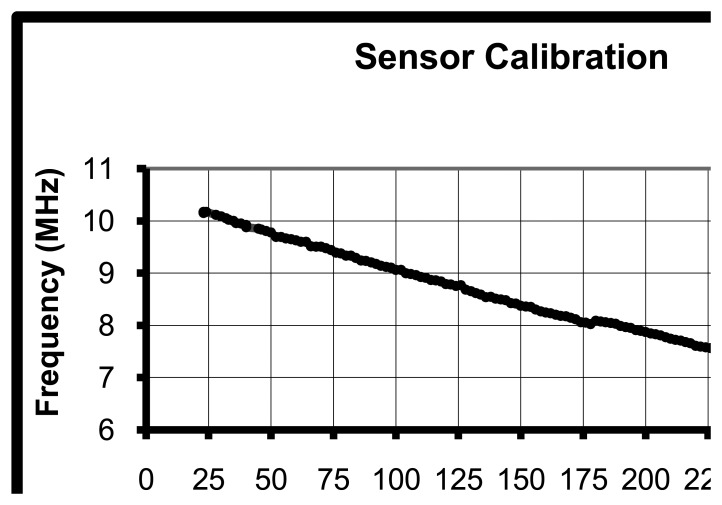
Sensor Calibration.

When the sweep frequency is generated by the Function Generator, the resonant frequency would be monitored by the Oscilloscope or read out by Laptop. Resonant frequency variations are recorded along with temperature variations. The calibration result is plotted in Figure 12.

This result proves that the *LC* temperature sensor made of this high-k ferroelectric material is feasible in wireless temperature monitoring. The ferroelectric material of the capacitor is responsive to environment temperature. This demonstrates that the capacitance is sensitive to temperature variations and then the resonant frequency is responsive to environmental temperatures.

## 5. Conclusions

This paper presents the development of a novel passive wireless temperature sensor, capable of operating in harsh environments for high temperature rotating component monitoring. A passive *LC* resonant telemetry scheme, relying on a frequency variation output, has been integrated with the sensor, thereby eliminating the need for physical contacts, active elements, or power supplies, which cannot withstand harsh environments. This project extended the knowledge base of passive wireless temperature sensing technologies in harsh environment and advance embedded prognostics health monitoring technologies. In conclusion, in this paper, the unique high-k temperature sensitive ceramic material has been applied instead of any electronic components to realize *LC* tank temperature sensing. A novel high-k temperature sensitive ceramic material has been developed which is able to work in harsh environment up to 235°C. Additionally, the unique temperature sensitive ceramic material has been integrated into the *LC* resonant tank circuit to create a frequency-encoded sensing mechanism which could dramatically increase sensitivity and dynamic response of the wireless temperature sensor.

## Figures and Tables

**Table 1.** System Parameter Values

Reader Inductance *L_R_*	1.5 μH
Sensor Inductance *L_S_*	0.68 μH
Sensor Total Resistance *R_L_*	6 ohm
Sensor Nominal Capacitance *C_S_* at 20°C	0.24 nF
Nominal Coupling Factor *k*	0.4
Reader Radius *r_R_*	30cm
Inductor Radius *r_S_*	28.5cm
Coupling Distance *d*	2.5cm
